# Synthesis and Performance Evaluation of a Novel High-Temperature-Resistant Thickener

**DOI:** 10.3390/molecules28207036

**Published:** 2023-10-11

**Authors:** Yu Sui, Tianyue Guo, Dan Li, Da Guo, Zhiqiu Zhang, Guangsheng Cao

**Affiliations:** 1 Key Laboratory of Enhanced Oil Recovery, Ministry of Education, College of Petroleum Engineering, Northeast Petroleum University, Daqing 163318, China; n2107778k@e.ntu.edu.sg (Y.S.); ldnepu0520@163.com (D.L.); zhang_zhiqiu@126.com (Z.Z.); 2 School of Mechanical and Aerospace Engineering, Nanyang Technological University, 50 Nanyang Avenue, Singapore 639798, Singapore; 3 China Petroleum Tarim Oilfield Branch Oil and Gas Engineering Research Institute, No. 26, Shihua Avenue, Korla 841001, China; 18646660514@163.com

**Keywords:** carbonate reservoir, temperature stability, polyacrylamide, spatial network structure

## Abstract

Successful exploitation of carbonate reservoirs relies on the acid-fracturing process, while the thickeners used in this process play a key role. It is a common engineering problem that thickeners usually fail to function when used in high-temperature environments. Until now, no research has ventured into the field of synthesizing thickeners which can be effectively used at ultra-high temperatures up to 180 °C. In our current study, a novel high-temperature-resistant polyacrylamide thickener named SYGT has been developed. The thermal gravimetric analysis (TGA) reveals that SYGT is capable of withstanding temperatures of up to 300 °C. Both our scanning electron microscopy (SEM) and rheological analysis demonstrate that the SYGT exhibits excellent resistance to both temperature and shear. At 180 °C, the viscosity of the SYGT aqueous solution is no lower than 61.7 mPa·s at a 20% H^+^ concentration or high salt concentration, and the fracture conductivity of the thickened acid reaches 6 D·cm. For the first time, the influence of the polymer spatial network’s structural parameters on the viscosity of polymer solutions has been evaluated quantitatively. It was discovered that the length and surrounding area of the SNS skeleton have a synergistic effect on the viscosity of the polymer solution. Our experiments show that SYGT effectively reduces the acid–rock reaction rate and filtration loss under harsh working conditions such as high temperature, strong shear, high salinity, and a high concentration of acid. The synthesized acid-fracturing thickener (SYGT) has wide application potential in the development of carbonate reservoirs under high-temperature conditions.

## 1. Introduction

Carbonate reservoirs hold 60% and 40% of the world’s total oil and gas reserves, respectively. To ensure their efficient development is vital for global energy security [1,2]. Acidizing fracturing, which constructs highly conductive fractures, can greatly boost oil and gas production [3,4,5,6]. The effectiveness of acidizing fracturing is evaluated by the effective action distance of acid and the conductivity of acid etch fracture [7,8,9,10,11]. However, conventional acidizing fracturing is hindered by the rapid reaction between hydrochloric acid and carbonate minerals, which results in significant wastage of hydrochloric acid in the near-well zone, while the far-well zone experiences poor acidizing fracturing effects [12]. A slow-release acid system has been developed to address this challenge by reducing the mass transfer coefficient of H^+^ and the acid–rock reaction rate by increasing the viscosity of the acid solution or chemically generating acid. This procedure can increase the effective acid action distance and allow the acid to penetrate deeper into the formation [13]. Gelled acid is a form of slow-release acid that exhibits good sand carrying, drag reduction, and filtration loss reduction properties [14,15,16,17,18]. Consequently, gelled acid is widely employed in the acid fracturing of carbonate rock.

**Figure 1:**
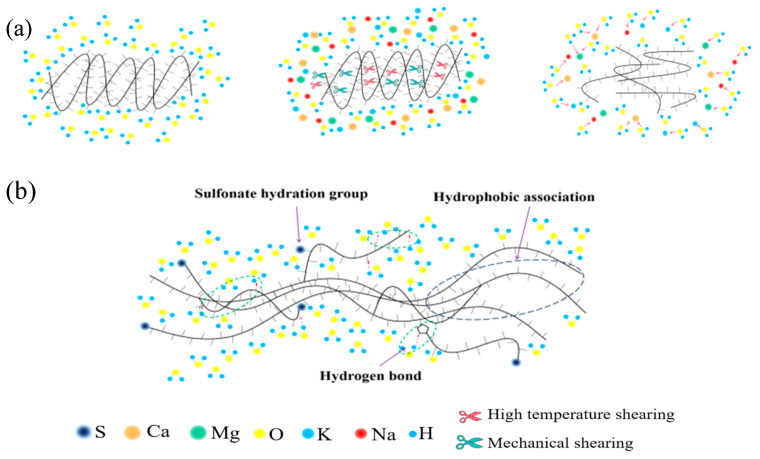
(**a**) Failure principle of thickener under high temperature, high salt and strong shear; (**b**) Schematic diagram of partial mechanism of temperature and salt resistance of synthetic SYGT.

The efficacy of thickened acid is dependent on the type of thickener used [19]. Thickeners are categorized as biological, cellulose, and synthetic polymers [20]. Biological polymers are unstable in high acid concentrations, and possess low temperature resistance, which limits their applicability [21,22]. Similarly, cellulosic-based polymers degrade easily, have poor shear resistance, and exhibit low temperature resistance, which make them unsuitable for use in oil fields [23]. Presently, the most widely used thickener is synthetic polyacrylamide [24,25]. However, with the increasing use of acidizing fracturing in high temperatures and deep wells in carbonate reservoirs, the amide group in polyacrylamide undergoes chemical degradation, causing molecular chains to shear, reducing molecular weight and hydrodynamic volume, and resulting in a decrease in viscosity, as seen in Figure 1a. Therefore, the viscosity of the thickened acid is significantly reduced under high-temperature conditions, resulting in a short effective acid action distance, a low conductivity of the acid-etched fracture, and a poor acid-fracturing effect. In this context, polyacrylamide’s temperature resistance poses new challenges. Additionally, as freshwater resources are scarce, using high-salinity brine or seawater directly to prepare acidifying fluids for fracturing has become a trend [26]. In deep formations, fluids typically have high salinity, which demands higher salt resistance from the acid solution. In addition, the adsorption of polyacrylamide in carbonate reservoirs also leads to a decrease in the concentration of effective components in the fracturing fluid, resulting in a decrease in viscosity [27,28].

In recent years, numerous studies have been conducted to improve the temperature and salt resistance of polyacrylamide [29,30,31,32]. Various strategies have been employed to enhance the temperature resistance of polyacrylamide, including the incorporation of rigid groups into the polyacrylamide backbone [33,34,35,36], such as sulfonic acid groups and cyclic materials, as reported in previous studies. Recently, a double quaternary ammonium salt copolymer has been synthesized [37], which exhibits remarkable temperature resistance and maintains a viscosity of 67 mPa·s at 170 °C. The results suggest that the incorporation of such groups into polyacrylamide molecules could improve their high-temperature stability, rendering them suitable for enhanced oil recovery (EOR) applications in harsh reservoir conditions. Despite the promising results obtained in those studies, further investigations are required to improve the experimental temperature and evaluate the viscosity of the system at an ultra-high ambient temperature, such as 180 °C, which has not yet been explored. Furthermore, research on the salt resistance of the system is limited. The high salinity, especially in the presence of divalent cations [38], has a significant impact on the electrostatic shielding effect [39], resulting in the entanglement of polymer molecules and weakening the viscosity-increasing effect. Moreover, the cations carried by polymers can disrupt the formation of worm-like micelles in the formation of water with high salinity, leading to a loss of viscoelasticity [40]. In this regard, some researchers have focused on improving the performance of crosslinking agents to enhance the stability of polymers. For instance, a covalent bond between polyethyleneimine and amide in NFT [41] can form crosslinking, which has been shown to impart superior temperature resistance to the resulting polymer solution. The development of various cross-linking agents to enhance polymer stability has been carried out, and the effectiveness of the developed agents has been verified. However, operational costs are also increased accordingly. Other researchers have focused on developing the polymer itself, such as hydrophobically associating polyacrylamide (HAPAM). They use acrylamide, acrylic acid, etc. as the main body, then modify them by introducing hydrophobic monomers to improve the linear flexible chain to a certain extent. A cationic hydrophobically associative polymer polyacrylamide [42] (C-HAPAM) has demonstrated good temperature and salt resistance under experimental conditions. The hydrophobic part incorporated into the main polymer chain is bonded in the water phase through intramolecular and intermolecular interactions to enhance the stability of the molecule. However, the experimental temperature of this study is relatively low, at 105 °C only. From the above discussion, it is apparent that there is a lack of research on developing a high-temperature-resistant polyacrylamide acid thickener. While there are several synthetic thickeners available for fracturing fluid, most polyacrylamide-based thickeners have poor acid resistance that has not been adequately investigated. This underscores the pressing need for a thickener that can withstand high temperatures and minimize viscosity loss when used in acidic fluids to address the current challenges associated with the thickening and acid fracturing of carbonate rocks.

In our current research, we have synthesized quaternary ammonium polymers based on AM (acrylamide), AMPS (2-acrylamido-2-methylpropanesulfonic acid), NVP (N-vinyl-2-pyrrolidone), and SSS (sodium polystyrene sulfonate). Additionally, we have synthesized ternary polymers using AM, AMPS, and SSS as starting materials for comparison purposes. The structures of the obtained polymers have been confirmed by infrared spectroscopy. The temperature and salt resistance mechanism of the copolymer are shown in Figure 1b. The rheological properties, temperature, salt resistance, and shear resistance of the polymer are evaluated through thermogravimetric analysis using rheometers and Brinell viscometers. The action mechanism of the spatial network structure (SNS) on the viscosity of the polymer solution is analyzed through SEM pictures and AutoCAD. The obtained viscosity of the polymer under acidic conditions is compared, and the mechanism of H^+^ action on the polymer is analyzed. Finally, the thixotropy, reaction kinetics, and filtration of the thickened acid system have been evaluated. The effect of the thickened acid is analyzed through experiments. Our investigation aims to propose a new type of high-temperature-resistant thickened acid-fracturing thickener, and explore the specific action mechanism of SNS on the viscosity of polymer solutions.

## 2. Results and Discussion

### 2.1. Synthesis and Characterization of SYGT

**Figure 2:**
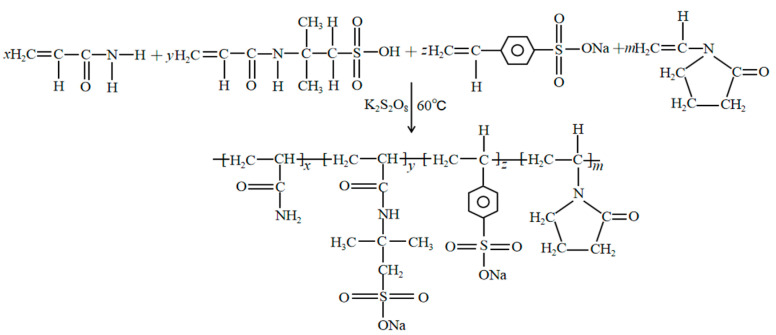
Schematic diagram of synthesis principle.

To design functional polymers, corresponding polymer synthesis experiments were carried out with acrylamide as the main chain, and monomers with rigid chain structures that exhibit high temperature and acid resistance as the side chain. The focus was not on maximizing yield but on achieving desirable properties. The formation of jelly-like gel indicates that the polymerization between the reaction monomers is successful, and a polymer with a viscosity-increasing effect is formed. With the progress of the reaction, the viscosity of the produced system gradually increases. The synthetic pathway is shown in Figure 2.

**Figure 3:**
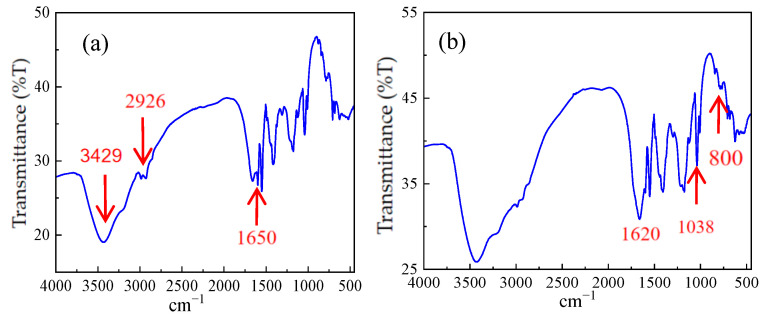
(**a**) Infrared spectrogram of ternary polymer; (**b**) Infrared spectrogram of quaternary polymer.

After squeezing the solid powder with a press, infrared mapping scanning was conducted using a Spectrum One FT-IR Spectrometer to verify the synthetic results of AM/AMPS/SSS polymer and AM/AMPS/NVP/SSS polymers, as depicted in Figure 3a and Figure 3b, respectively.

Generally speaking, 3429 cm^−1^ is the characteristic absorption peak of free −NH_2_, 3216 cm^−1^ is the characteristic absorption peak of associated −NH_2_, 2926 cm^−1^ is the characteristic absorption peak of the methylene antisymmetric stretching vibration, and 2850 cm^−1^ is the characteristic absorption peak of the methylene symmetric stretching vibration. The infrared spectra reveal that the characteristic peak of amide II (N−H bending vibration) is located at 1620 cm^−1^, the strong absorption peak around 1650 cm^−1^ is attributed to the amide group, and the absorption peak at 1038cm^−1^ is associated with the sulfonic acid groups. Furthermore, the characteristic absorption peak of the p-disubstituted benzene appears at 800 cm^−1^. A significant increase in peak intensity at 1561cm^−1^, corresponding to the secondary amide N−H bending vibration, was observed after cationization, indicating that N−H in polyacrylamide was replaced by N−R. Additionally, the peak at 1174 cm^−1^ is common in cationized products; it is generally related to C−N, and confirms that both the ternary and quaternary polymers synthesized are cationic polymers.

### 2.2. TGA Results and Analysis

**Figure 4:**
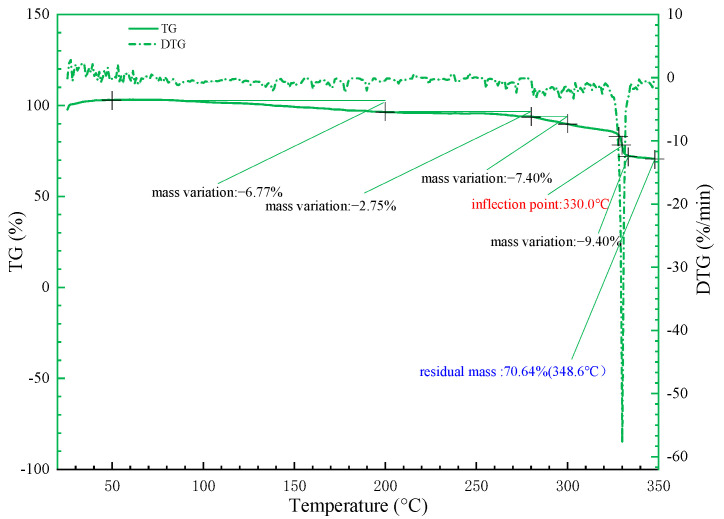
SYGT thermogravimetric curve.

The decomposition temperature of polymers mainly depends on the thermal stability of macromolecular groups. The introduction of the sulfonic acid group and benzene rings leads to an increase in the molecular weight of SYGT, and greatly enhances the temperature resistance of SYGT. The TGA (thermogravimetric analysis) and DTG (differential thermogravimetric analysis) curves of SYGT are shown in Figure 4.

Thermogravimetric analysis was conducted to evaluate the thermal stability of the polymer in the temperature range of 25−350 °C. The polymer exhibited relatively stable thermal decomposition at the experimental temperature, with a noticeable decline in mass occurring at 330 °C.

There was no obvious mass loss of the polymer at 25−100 °C, indicating that the sulfonic acid and acrylamide groups in the polymer had already removed the adsorbed water in the drying chamber. At 280−320 °C, the polymer mass decreased relatively significant, at 7.4%. The most significant mass loss of 9.4% occurred near 330 °C. The DTG curve indicated that the change was the largest at this temperature, and the final (348.6 °C) mass residual rate was 70.64%. In order to verify the thermal stability of the polymer, the study results of Zhang [43] are compared with the current thermal stability of SYGT. At about 350 °C, the polymer weight loss rate studied by Zhang is generally lower than 70%, around 60%. A comparative analysis of the thermal stability data proves that SYGT has better thermal stability.

### 2.3. Rheological and Temperature Resistance Test

**Figure 5:**
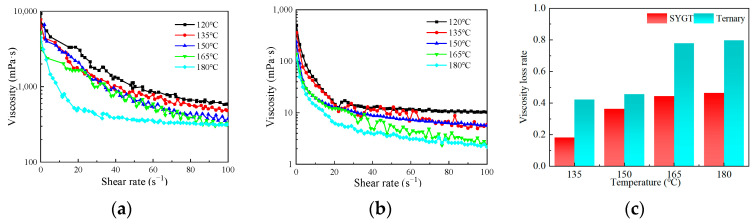
(**a**) Rheology of SYGT; (**b**) Rheology of ternary polymers; (**c**) Viscosity loss rate at different temperatures.

The rheological properties of ternary polymers and SYGT at 120, 135, 150, 165 and 180 °C are shown in Figure 5a and Figure 5b, respectively. The corresponding viscosity loss rates at different temperatures are shown in Figure 5c.

It can be seen from Figure 5a,b that both the rheology of terpolymer and SYGT decrease as the temperature and shear rate increase. The molecular chain of polyacrylamide is broken with increasing temperature, resulting in shorter molecular chains and the destruction of the spatial network structure of the polymer. The molecular hydrodynamic volume becomes smaller, and the viscosity of the polymer solution decreases macroscopically. Similarly, as the shear rate increases, the mechanical shear effect becomes more pronounced, resulting in a shorter molecular weight and a decrease in the polymer viscosity. In comparison to the viscosity of the existing thickeners at 180 °C, which is approximately 50 mPa·s, SYGT exhibited a viscosity of 316 mPa·s, and demonstrated remarkable viscosity-increasing and temperature resistance properties.

The viscosity of SYGT at 180 °C decreased by 46.45%, while the terpolymer showed a greater decrease of 79.82% compared to that at 120 °C, as depicted in Figure 5c. It should be noted that the viscosity average molecular weight of the synthesized ternary polymer is 8.23 × 10^5^, and the molecular weight of SYGT is 8.57 × 10^5^. The difference in molecular weight between the two is not significant, although SYGT is a quaternary copolymer. This result indicates that SYGT has superior temperature resistance compared to the terpolymer. In addition, the viscosities of SYGT and the terpolymer are significantly different. Under a shear rate of 180 °C and 100 s^−1^, the viscosity of SYGT remained 316.52 mPa·s, indicating a remarkable viscosity-increasing effect.

**Figure 6:**
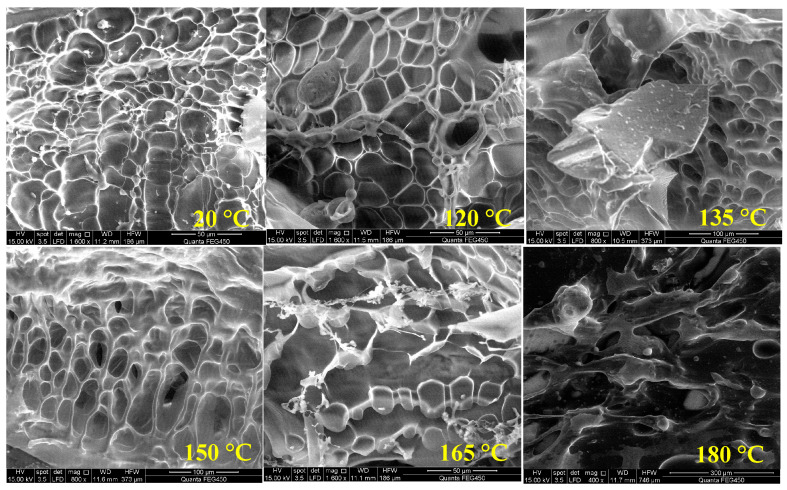
Polymer morphology at different temperatures.

To explore the temperature resistance mechanism of the polymer, its morphology was studied using scanning electron microscopy for the polymer solutions at temperatures of 20 °C, 120 °C, 135 °C, 150 °C, 165 °C, and 180 °C, respectively, as shown in Figure 6.

At 20 °C, a distinctive “fish-scale” spatial network structure (SNS) is visible in the polymer solution under SEM. With increasing temperature, the SNS is progressively disrupted. The local “fish scales” are fractured and no longer appear as a cohesive structure, particularly at 165 °C and 180 °C. The hydration structure formed by the polymer molecules is destroyed, and the hydrodynamic volume is reduced.

**Figure 7:**
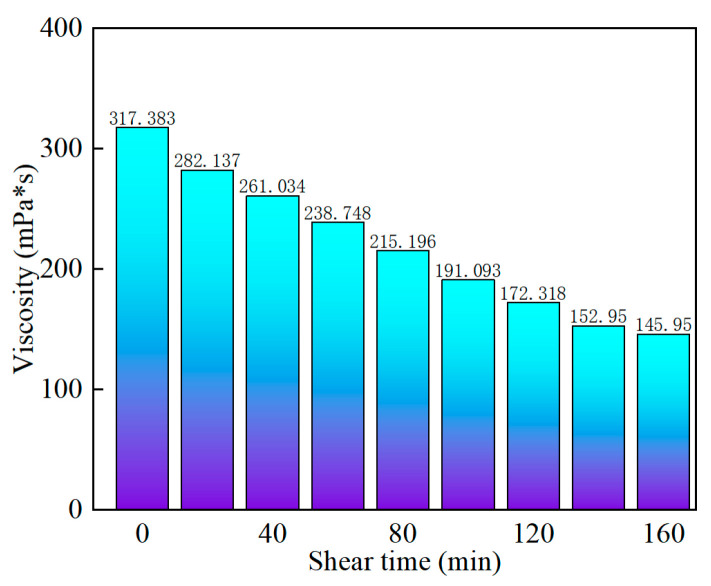
Variation in polymer viscosity with shear time.

In order to explore the shear resistance of SYGT at high temperatures, a shear test was performed at 170 s^−1^ and 180 °C for 160 min, respectively. The experimental results show that the viscosity loss rate is 54.01%, and the viscosity is 145.95 mPa·s. It can be seen from the data that SYGT has competent shear resistance under high-temperature conditions, as shown in Figure 7.

**Figure 8:**
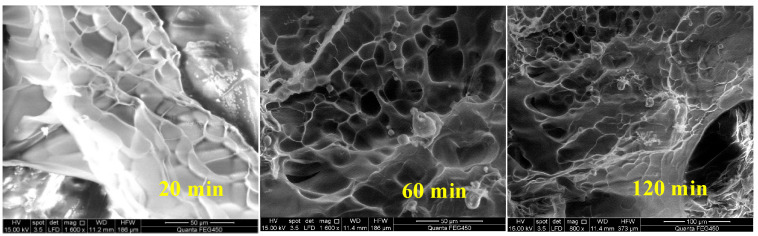
Morphology of polymer solution after shearing at different time.

As the shear time increases, the viscosity gradually decreases and tends to reach a stable state, indicating that the establishment and destruction of the polymer’s spatial network structure have reached a balance, which is proven in Figure 8. After shearing the polymer for 60 min, compared with the case of 20 min shearing, the degree of SNS damage is greater. After 120 min of shearing, the damage degree is slightly increased compared with the case of 60 min of shearing, but the SNS is basically stable.

### 2.4. Effect Mechanism of SNS on Viscosity

Based on our current research, it is concluded that temperature affects the size of polymer molecules through the “high-temperature shear” of polymer chain, leading to changes in the spatial network structure of polymer aqueous solutions and ultimately affecting the viscosity of the solution. However, the impact of specific parameters related to the distribution of the polymer’s spatial network structure on the solution’s viscosity remains understudied. Just like the trunk and branches of a tree, the density and thickness will affect the distribution of leaves, and the leaves will affect the flow of air. The distribution width of the polymer solution space network framework is also fundamental to the formation of a polymer control body by the polymer-constrained water molecules. However, the distribution and skeleton width of the polymer molecules under different temperatures, concentrations, and shear conditions of a shear body are different, thus showing different levels of aqueous solution viscosity.

**Figure 9:**
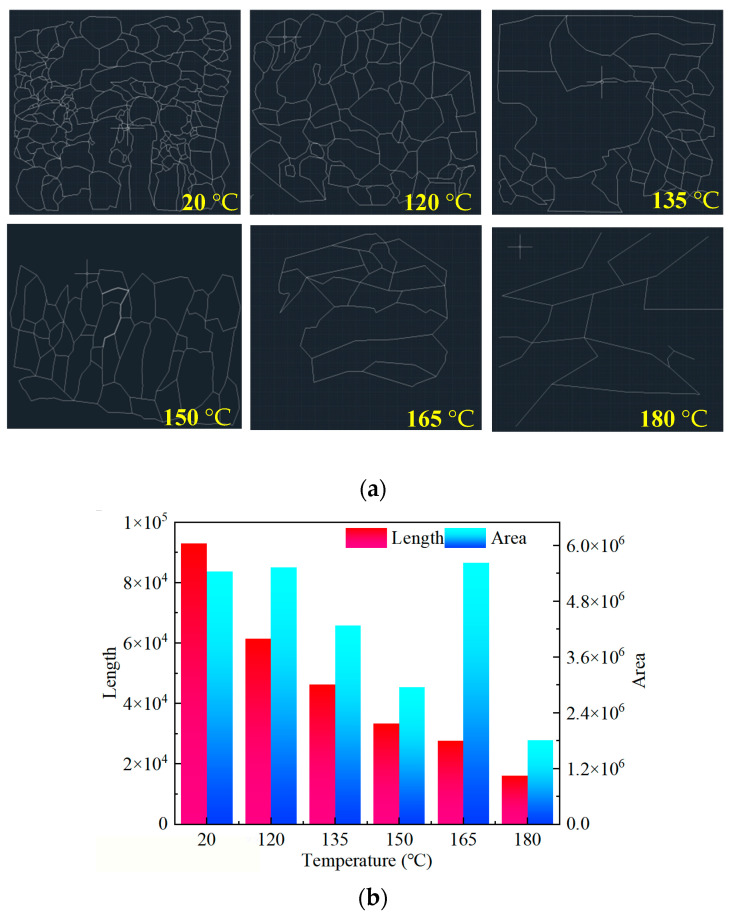
(**a**) SEM image of the SNS skeleton; (**b**) Length and area statistics of the SNS skeleton.

The SEM image presented in Figure 6 is processed using AutoCAD 2023 software to obtain the skeleton line of the SNS, as shown in Figure 9a.

The length and surrounding area of the SNS skeleton are calculated using AutoCAD. The magnification of the picture is inconsistent during processing, but the statistical results of AutoCAD, including the length and area, are made to be dimensionless, which eliminates the impact of inconsistent magnification. The statistical results are shown in Figure 9b.

It is seen from Figure 9b that the skeleton length of SNS decreases with increasing temperature, and its value at 180 °C is only 17.27% of that at 120 °C. The polymer molecular chain is continuously sheared with increasing temperature. The control ability of the fluid is continuously reduced, which leads to continuous reduction in the skeleton length of the airborne network structure. The overall distribution also continuously becomes sparse. From a two-dimensional perspective, the utilization of the plane is reduced, as shown in Figure 9a.

The are surrounding the skeleton also shows a negative correlation with the increase in temperature. However, there was a certain increase at 165 °C. The drawn SNS skeleton is basically composed of closed lines. Although the enclosing area is large at 165 °C, as can be seen from Figure 6, the actual fluid control in the area of the polymer is not the same as in its enclosing area. Therefore, the enclosing area at 165 °C is overestimated in the calculation process. This also indicates the existence of a certain synergistic effect between the length and enclosing area of the SNS. Even if the SNS area is large enough, it still requires a long enough skeleton to support it, and a reasonable spatial layout is needed; otherwise, it is difficult to form a continuous and effective water molecule control body.

From the above analysis, it is evident that the length and surrounding area of the SNS skeleton have a synergistic effect on the viscosity of the polymer solution. Therefore, in future research, it will be necessary to refine the influence of the SNS skeleton parameters on the viscosity of the polymer solution. This includes expanding the parameters, quantifying their effects, and ultimately deriving a consensus on the relationship between the SNS skeleton and the viscosity of the polymer solution.

### 2.5. Acid and Salt Resistance Test

**Figure 10:**
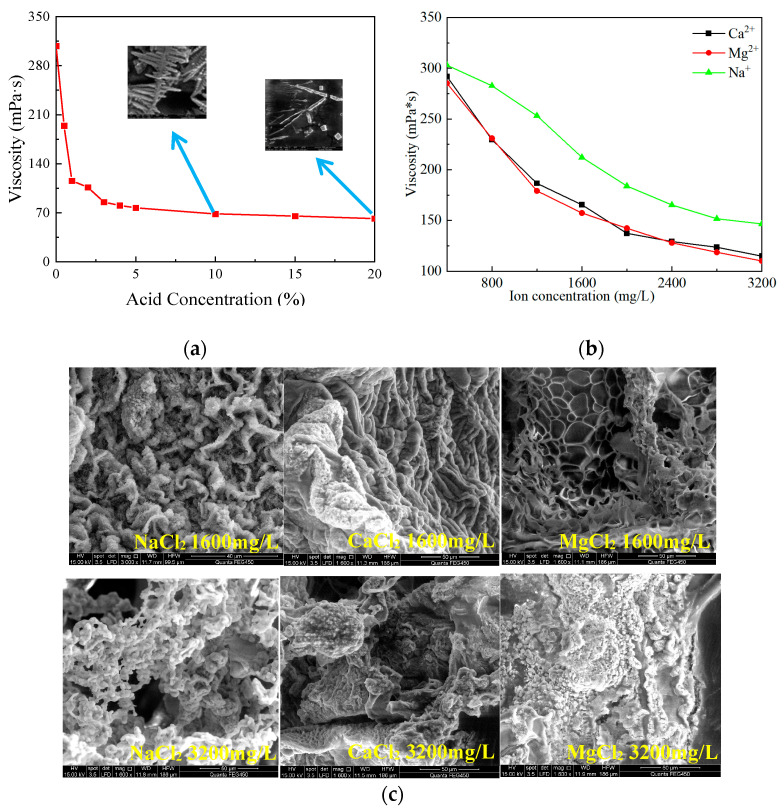
(**a**) Variation in viscosity with acid concentration; (**b**) Viscosity verses inorganic salt concentration; (**c**) SEM images of SYGT under different conditions.

The acid concentration used on the field is typically 20%, but with the reaction between acid and rock, the acid concentration gradually decreases. To investigate the effect of acid concentration on the viscosity of SYGT solution, 1 g of the SYGT was dissolved in 100 mL acid solution with varying concentrations. The upper limit of acid solution concentration is set at 20%. The viscosity was measured at 180 °C with a shear rate of 170 s^−1^. The results showed that the viscosity of SYGT solution decreased with the increasing acid concentration, and then leveled off. At an acid concentration of 20% HCl, the solution viscosity of SYGT was 61.7 mPa·s, as shown in Figure 10a. Hydrogen ions induce the crimping of the molecular chains of SYGT, leading to an improvement in hydrogen bond interaction (within the molecules) and a reduction in the hydrodynamic radius. Additionally, the oxidation of H^+^ attracts the electrons in the shared electron pair, thereby affecting the stability of the shared electron pair. From Figure 10c, the spatial structure was significantly damaged, changing from mesh to rod, resulting in a decrease in the polymer viscosity.

As mentioned in the introduction, due to a shortage of fresh water resources, it has become a trend to prepare acid solutions with seawater. To evaluate the salt resistance of SYGT, the salinity (0.32 g/L) of the seawater in Liaodong Bay of Bohai Sea [44] was taken as the upper limit.

The viscosity of the SYGT solution gradually decreased and finally stabilized with the increasing inorganic salt ion concentration, as seen in Figure 10b. High-valence calcium and magnesium ions act as charged salt ions that shield the electrostatic repulsion between SYGT molecules, causing the macromolecular chain to curl. The dehydration of inorganic salt ions removes the hydration water from the copolymer chain, reducing its hydrodynamic volume.

The lowest viscosity values (110.1 mPa·s, 114.88 mPa·s, and 146.59 mPa·s) were obtained with calcium salt, magnesium salt, and sodium salt concentrations of 3200 mg/L, respectively. The corresponding viscosity loss rates were 61.4%, 60.65%, and 51.63%. These results indicate that the SYGT solution maintains a high viscosity even under high inorganic salt concentrations, with a relatively low viscosity loss rate.

### 2.6. Kinetics of Reaction

**Figure 11:**
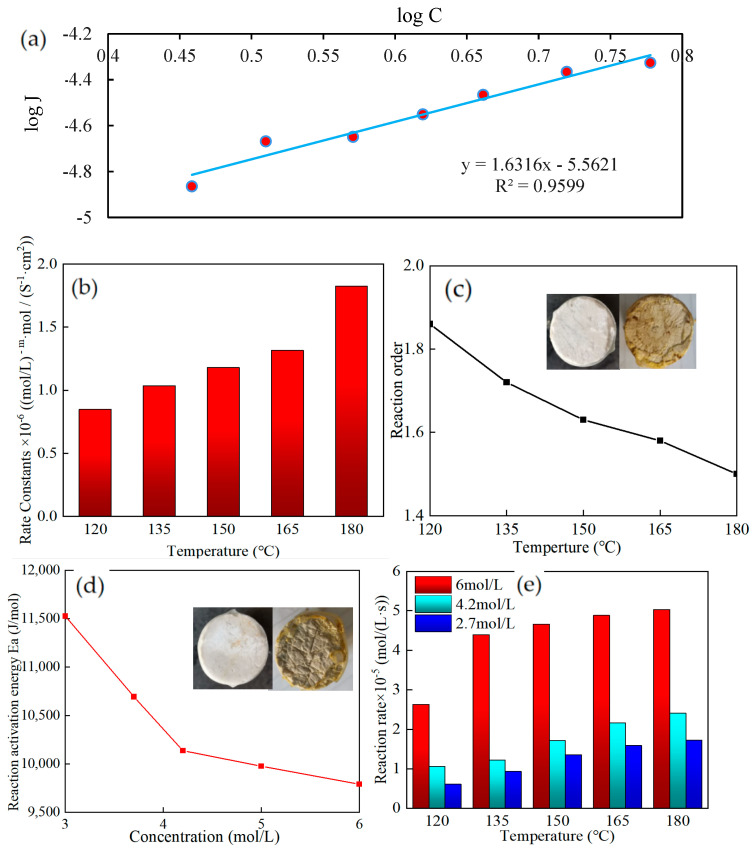
(**a**) Reaction kinetics parameter simulation at 150 °C; (**b**) Rate constant; (**c**) Reaction order; (**d**) Reaction activation energy; (**e**) Reaction rate.

Using a rotating disk reaction system in a dynamic environment, the kinetic equations of the acid–rock reaction at various temperatures (120–180 °C) and acid concentrations (2.7–6 mol/L) were determined. The speeds of acid–rock reactions at various temperatures were contrasted. As an illustration, the data fitting of reaction kinetic parameters at 150 °C was used in Figure 11a. The J in the figure refers to the diffusion rate, which is the number of moles of material flowing through unit rock area per unit of time. The fitting effect was fair, and the fitting precision was 0.9599.

The rate constant of the thickened acid solution added with SYGT and carbonate rock increased with increasing temperature, as shown in Figure 11b. The reaction order decreases with the increase in temperature, as shown in Figure 11c. Compared with 1.15 reaction order of VES at 120 °C [45], the reaction order of SYGT was 1.8 at 120 °C from the reaction results. At 180 °C, the acid–rock reaction order was 1.5, the rate constant was 1.82, and the reaction rate of 6 mol/L was 5.03 × 10^−5^ mol/(L·s). The thickening acid effectively controlled the reaction.

According to the physical meaning of the reaction order, the larger the reaction order, the greater the effect of concentration on the reaction rate [46]. The reaction order of the thickened acid added to SYGT is higher than that of VES, which indicates that the acid concentration has a more obvious influence on it. The concentration of the acid solution increases the collision probability of the activated molecules. The more dependent it is on the concentration, the better its effect in slowing down the reaction.

The Arrhenius formula was used to determine the acid–rock reaction activation energy, as illustrated in Figure 11d. The graphic shows that as the acid concentration increases, the activation energy of the acid–rock interaction falls. The normal theory states that the activation energy of a reaction is a physical property that is independent of the concentration of the reactants. The acid–rock reaction, however, is an exothermic reaction, meaning that as it proceeds, a significant quantity of heat is generated, warming the surrounding area and increasing the acid–rock reaction. The acid concentration and the temperature cause the reaction to proceed in a positive-feedback way. The reaction speed is accelerated, as shown in Figure 11e.

### 2.7. Filtration of SYGT

**Figure 12:**
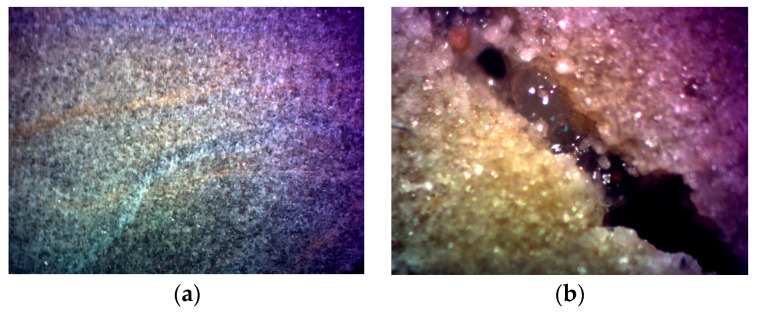
(**a**) Core before experiment; (**b**) Acid in the core and fractures after the experiment.

Experiments were carried out to study the effect of static fluid loss on the thickening of acid over a temperature range of 120−180 °C. Linear regression analysis was performed on the experimental data to obtain the slope of the curve, and the parameters *C*_3_ and *v_c_* in Equations (6) and (7) were calculated under different temperature conditions. The core before and after the experiment is shown in Figure 12. The core after the experiment clearly demonstrates the dissolution fractures that have undergone filtration and the acid solution that is being filtered. At 180 °C, the filtration coefficient and filtration velocity of the thickened acid were 0.1747 × 10^−4^ m/min^0.5^ and 0.2912 × 10^−5^ m/min, respectively. It can be seen from the calculation results in Table 1 that the filtration loss of the acid solution under high-temperature conditions is well controlled.

### 2.8. Evaluation of the Conductivity of Acid-Etched Fractures

**Figure 13:**
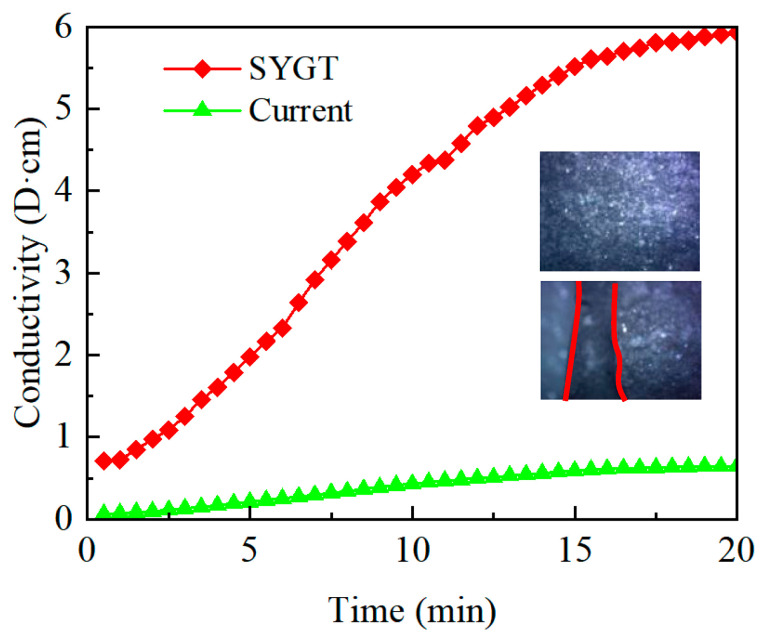
Conductivity of the thickened acid-etched fractures.

An acidizing fracturing conductivity tester was used to measure the acidizing fracturing conductivity of the natural core samples of Tadong Oilfield after being dissolved for 20 min using the thickened acid (10 mL/min injection rate, 180 °C ambient temperature). The measured results were compared with those of the field thickener after acidizing fracturing. The experimental results are shown in Figure 13.

It can be seen from Figure 13 that after the SYGT-thickened acid dissolution, there are obvious acid-etched wormholes. In addition, the fracture conductivity of the SYGT-type thickened acid after dissolution is significantly higher than that of the on-site thickened acid, with the highest conductivity reaching 6 D·cm. The etching effect is significant after acid fracturing.

## 3. Material and Methods

### 3.1. Materials

AM, AMPS, and NVP were sourced from Shanghai McLean Biochemical Technology Co., Ltd. (Shanghai, China). SSS was purchased from Beijing Datian Fengtuo Co., Ltd. (Beijing, China). Analytically pure reagents including NaOH, NaCl, CaCl_2_, and MgCl_2_ were provided by Tianjin Yongda Chemical Reagent Factory, Tianjin, China. Deionized water was self-made in the laboratory. All chemicals and reagents were used without further purification.

### 3.2. Synthesis of SYGT

In order to solve the problem of acid thickener failure under high-temperature conditions, AM, AMPS, NVP and SSS were used for polymerization in a water bath. The monomer ratio was designed to be 4AM:1AMPS:0.3NVP:0.3SSS for quaternary polymerization and 4AM:1AMPS:0.3SSS for ternary polymerization.

AM, AMPS, and NVP were mixed in deionized water, and NaOH solution was used to adjust the pH to 6 after stirring evenly. The temperature of the water bath was set at 40 °C. The rate of the agitator was adjusted to 300–350 r/min, and nitrogen was used to remove oxygen. After that, the temperature was set at 60 °C. During the heating process, K_2_S_2_O_8_ was added to the three-neck flask. After the temperature reached 60 °C, the rotating rate was set at 150 r/min. After 2 h, a certain amount of SSS monomer was added to the flask and reacted for 12 h. Finally, the solution was purified in ethanol and dried in a vacuum for 12 h in an oven at 50 °C.

### 3.3. Polymer Performance Evaluation

#### 3.3.1. Determination of Viscosity Average Molecular Weight

**Figure 14:**
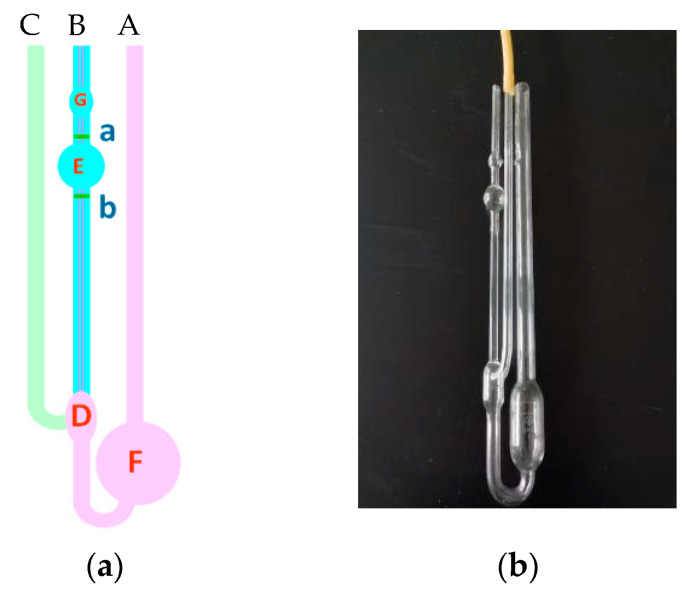
Schematic diagram of the Ubbelohde viscometer.

(1) A total of 15 mL of the prepared 1000 mg/L polymer solution should be drawn with a pipette; it should be injected from viscometer tube A in Figure 14, and the temperature should be kept at 30.05 °C for 5 min.
(2) After constant temperature for 5 min, tube C should be pressed by hand, the ear ball should be placed at the mouth of tube B, the solution should be drawn into ball G, and it should be released.
(3) When the solution drops to line a, the time should be recorded by pressing the stopwatch, and when the solution drops to line b, the experiment should be ended by pressing the stopwatch.
(4) The measurement should be repeated three times, and the time difference between each time should not exceed 0.2 s.
(5) After measuring the polymer solution with a concentration of 1000 mg/L, 1 mol/L NaCl solution should be sucked into the volumetric flask in a constant temperature bath using a pipette, and it should be added from tube a. Tube C should be pressed and held, and the solution should be repeatedly pressed and sucked from the orifice of tube B using a suction ear ball to make the mixture uniform.
(6) The determination method is shown in (3) and (4).
(7) Some 2 mL, 2 mL, and 4 mL of 1 mol/L NaCl solution should be added successively, and measured according to steps (5) and (6).
(8) The relative viscosity of the sample solution should be calculated according to Formula (1):
  (1) $$\eta_{r}=\frac{t}{t_{0}}$$

where: *η_r_*—relative viscosity; 

*t—*flow time of sample solution, s;

*t*_0_*—*flow time of 1 mol/L sodium chloride solution, s.

The corresponding [*η_r_*]·*C* value can be found from the obtained *η_r_*, and the [*η_r_*]·*C* value can be divided by the sample concentration *C* to obtain [*η_r_*].

(9) The molecular weight is calculated according to Formula (2):

(2) $$[\eta ]=KM^{\alpha }$$

where: *M*—average value of relative molecular weight of polymer;

*K*—proportional constant, *K* = 3.34 × 10*^−^*^4^;

*α*—empirical parameters related to the morphology of polymers in solution, *α* = 0.708.

#### 3.3.2. Thermogravimetric Analysis (TGA)

To investigate the thermal stability of SYGT, TGA experiments were conducted using STA449 F5 Jupiter, which is manufactured by NETZSCH in Selb, Germany). Nitrogen was used as both the purge gas and the protection gas at a flow rate of 60 mL/min. The temperature of SYGT was increased at a rate of 10 °C/min, ranging from 25 °C to 350 °C.

#### 3.3.3. Infrared Spectrum Scanning

To confirm the successful synthesis of polymer SYGT, infrared scanning experiments were conducted using a Spectrum One FT-IR Spectrometer.

After the KBr powder was dried, 1 spoonful of SYGT solid was mixed with 3 spoonfuls KBr powder, and the two were uniformly mixed by grinding in a mortar. Finallythe powder was pressed with a punch and scanned with the Spectrum One FT-IR Spectrometer.

#### 3.3.4. Rheological Testing and Viscosity Testing of SYGT Aqueous Solution

The current thickener is ineffective when the viscosity keeps increasing at high temperatures. To investigate the physical mechanism of the phenomenon, the viscosity of its aqueous solution was measured using a HAAKE RheoStress 6000 (Thermo Fisher: Waltham, MA, USA) rotating rheometer at high temperatures (120–180 °C) under varying conditions, such as different shearing times and properties. The viscosity-increasing effects of both the SYGT and ternary polymers (AM/AMPS/SSS) were compared at high temperatures.

#### 3.3.5. Reaction Kinetics of SYGT

The acid–rock reaction was conducted in a rotating disk system at temperatures ranging from 120 °C to 180 °C to determine the kinetics and activation energy of the reaction. This experiment aimed to investigate the reaction rate of the acid and rock with the synthetic thickener, and to explore the retarding effect of SYGT.

Data processing method:

In the processing of the actual test data, the reaction speed after correction of the face volume ratio is adopted:

(3) $$J=-(\frac{\partial C}{\partial t})\frac{V}{S}$$

Its principle formula is as follows:

(4) $$J=kC^{m}$$

The *C* value and *J* value of Equation (2) can be measured under a certain temperature, pressure, and rotating speed using a rotating disc device. The acid rock reaction rate is determined using a differential method, and the relation curve is drawn:

(5) $$J=\left(\frac{C_{2}-C_{1}}{\Delta t}\right)\frac{V}{S}$$

For Formula (3), we obtained the following:

(6) lgJ=lgk+mlgC

The reaction rate constant *k* and the reaction rate order *m* are constants under certain conditions. Therefore, a straight line is drawn using lg*J* and lg*C*, and the linear regression of lg*J* and lg*C* is carried out using the least-squares method to obtain the values of *k* and *m*, so as to determine the acid–rock reaction kinetic equation.

#### 3.3.6. Static Fluid-Loss Test

At 120 °C, 150 °C, and 180 °C, static filter loss tests were performed, respectively, using six-link water loss equipment.

The filter loss factor is governed by the filter cake (*C*_3_), and the filter rate (*v_c_*) can be determined after sketching the static filtration curve of the fracturing fluid using Equations (7) and (8).

(7) $$C_{3}=0.005\times \frac{m}{A}$$

(8) $$\nu_{c}=\frac{C_{3}}{\sqrt{t}}$$

where: *C*_3_—filter loss factor controlled by filter cake, *m*/$\sqrt{min}$;

*m*—the slop of the filter loss curve, $mL/\sqrt{\min}$;

*A*—the filtration area, cm^2^;

*v_c_*—the filter rate, m/min;

*t*—the filtration time, min.

## 4. Conclusions

The synthesized SYGT is a cationic polyacrylamide with quaternary ammonium salt side chains. After conducting various experimental tests and analyzing the obtained results, the following conclusions may be drawn:

(1) SEM and rheological analysis indicate that SYGT has good temperature and shear resistance. At 180 °C (100 s^−1^), the viscosity of the SYGT aqueous solution is 316.52 mPa·s. Even after 160 min of shear, it still maintains a viscosity of 145.95 mPa·s. The thermogravimetric analysis shows that SYGT can withstand high temperatures up to 300 °C.
(2) At 180 °C, the SYGT aqueous solution still has a minimum viscosity of 61.7 mPa·s at a 20% H^+^ concentration or high salt concentration. The reaction kinetics test proves that the SYGT-thickened acid effectively controls the reaction.
(3) It was found that the polymer spatial network parameters have a substantial influence on the viscosity of polymer solutions. For the first time, the length and surrounding area of the SNS skeleton have been quantified. Our research demonstrates that there is a synergistic effect of the length and surrounding area on viscosity.

The synthesized SYGT has good temperature resistance, acid resistance, salt resistance, and shear resistance. SYGT can effectively fill the gap in the high-temperature field of carbonate-rock-thickening and acid-fracturing technology. In our future work, we will further explore the influence of SNS on the viscosity of polymer solutions.

## Figures and Tables

**Table 1:** Filtration coefficient and filtration rate.

Temperature	120 °C	135 °C	150 °C	165 °C	180 °C
Filtration coefficient × 10^−4^ (m/min^0.5^)	0.1031	0.1187	0.145	0.165	0.1747
Filtration rate × 10^−5^ (m/min)	0.1718	0.1978	0.2417	0.275	0.2912

## Data Availability

Not applicable.
